# Proceedings of Maryland and District of Columbia Dental Association. Annual Meeting

**Published:** 1881-03

**Authors:** 


AMERICAN JOURNAL

OF

DENTAL SCIENCE.

Vol. XIV. THIRD SERIES—MARCH, 1881. No. 11.

### ARTICLE I.

### *Proceedings of the Maryland and District of Columbia Dental Association.—Annual Meeting.*—(Continued.)

Second Day,

*Morning Session.*

The morning hours were devoted to clinics.

---

*Afternoon Session.*

The afternoon session was called to order at two o'clock by the President.

The minutes of the previous meeting were read and approved.

Dr. Winder proposed as members of the Association, Drs. L. C. Hugo, of District of Columbia; Edward Flannigain, of Maryland; M. F. Thompson, of District of Columbia, all of whom were unanimously elected.

Dr. Waters moved that a committee of six be elected from the Association to provide, at times, suitable articles for the press that will tend towards the enlightenment of the public. He suggested that all articles written for the press be first referred to the committee for their approval. The motion was carried.

A card was read from Dr. H. H. Keech, tendering his resignation as a member of this Association. Accepted.

An advertisement of Dr. Wm. A. Mills, of Baltimore, was read and charges prefered. The President appointed Drs. T. W. Coyle, E. P. Keech and T. S. Waters a committee to confer with Dr. Mills.

Reading of papers and discussions being in order, Dr. W. W. Evans, of Washington, D. C., chairman of the section on Artificial Dentistry read the following paper:

*Gentlemen of the Dental Association of the State of Maryland and the District of Columbia:*

I am about to do for my section what I have so frequently criticised in others—slight it. Owing to ill health in the past year and inability to get assistance from some members of my section whom I counted upon, I am left really without material to work from, unless I make a "rehash" of what I have already laid before this Association in past years.

I have advanced the principles frequently of making Artificial Dentistry a specialty, and of administering practical, educational facilities to students in our college; and I am now more convinced than ever of the necessity of such a step, since visiting abroad and noting the primitive state of conducting this branch. And yet I saw manipulative skill (jewelry work) that had it been intelligently, artistically directed, and the *workman* allowed entirely (that is to say, handle the patient as well as the plate) would have produced marvelous results.

I am glad to note that in the past two years we have advanced in this branch very materially. The manufacturers are meeting us more than half way, and the colleges have gotten so far as to make more special notices in their catalogues.

A valuable acquisition to our dental literature, and particularly so in Dental Art, is Dr. Kingsley's late work entitled "A treatise on Oral Deformities as a branch of

Mechanical Surgery." Although practically deficient in many points and slightly arrogant in tone, it is nevertheless very rich in valuable matter connected with Artificial Dentistry. It will give food for thought, especially so by dwelling on the chapter devoted to "Aesthetics of Dentistry," and should be found in the possession of every dental student as well as in the library of all lovers of Esthetic Dental Prosthesis. We have too few works of this kind, to afford to throw them aside without notice. I also understand, that there is just out a revised edition of "Richardson's Mechanical Dentistry." As I have not seen it, I cannot criticise its merits or demerits.

We have among the improvements and matters new to the profession in the past year—Dr. Campbell's New Mode Heater for Celluloid or Vulcanite, Dr. J. B. McPherson's Articulator, and a compound of pink wax and paraffine, rolled very thin for making up base plates, by the firm of S. S. White. They are all valuable acquisitions in their way to one neglected branch, and go to show the marked improvement of our profession.

The first is likely to establish the permanent claims of Celluloid, which is fast taking the place of Vulcanite. As we learn to manipulate it more thoroughly, with the advantages possessed by Dr. Campbell's apparatus, viz., the peculiar dry heat produced by steam, which makes it almost impossible to burn your plate; (wherein lies one of its greatest troubles for durability) preserves the color much better; produces a harder surface and is less liable to warp, we must succeed in time in producing a long needed base material, where single teeth alone will be required, for it is to be heartily wished for, that the day is not far distant when what is known as block teeth, or sectional gum teeth, will be entirely dispensed with. Not but what they can be produced to closely imitate nature, but the extra delicacy of carving and trimming, the number of blocks destroyed by warping, &c., &c., would compel the manufacturers to ask more than the ninety-nine out of the hundred dentists would be willing to pay for; thereby making the sales unprofitable. Then again, they are scarcely applicable for anything but full cases, whereas the single teeth admit of the most artistic arrangement, approximating nature so closely, by skillful manipulation, as to defy detection in the mouth of the most critical observer. The improvements also in this class of teeth have been very decided of late.

I had hoped to show some specimens at this meeting, of gold and platina plates in combination with celluloid, that I believe is entirely new to the profession, which we will have to put off for the present, as I have not had time to get them up. With rubber also, celluloid makes strong, durable work and very beautiful.

The wax mentioned above for base plates is wonderfully tough and pliable, and is just the thing we want in manipulation of celluloid; being semi-transparent and of a shade approximating the natural gum allows me to see the lines of the mouth on the model and admits of fine carving.

Last though not least, is the subject of pivot teeth. Building up contour gold crowns with foil, and putting on crowns of swedged plate metal, &c., may be very pretty for dentists to admire, but I think, that we can simplify, as well as beautify our patients mouths if we can mount upon the old roots, porcelain crowns of some kind, to more closely imitate their articulating neighbors.

People are becoming tired of these long tedious operations, as well as unwilling to pay the fee we ought to receive for a well executed contour gold crown. What is known as the Richmond plan is quickly executed so far as the patient is concerned, but has many drawbacks, not least among them is their unsightliness. An article by Dr. W. G. A. Benwill, published in the August *Cosmos* of this year contains some valuable suggestions, worthy of discussion.

In closing I can only tender an apology for this hastily put together and abruptly ended paper.

Under this head Dr. Reese, of Brooklyn, exhibited some specimens of artificial work made on gold alloy base, and explained the manner of working the same.

Dr. Atkinson said the main feature of the gold alloy base is, the relation it holds to the conductivity of the tissues. He thinks it the best material in use for dental plates. Artificial dentistry was then passed.

Dr. Winder read a paper on food stuffs as a power, which was read by Dr. Cutter, of Boston, at the meeting of the Association held in Baltimore, in 1879, [see Am. Jour. of Dental Science, Jan. 1880, p. 407,] and discussion postponed until this meeting because of a want of time to do it justice. After which the meeting adjourned until the evening session.

---

*Evening Session.*

The Association was called to order by the President.

Dr. Atkinson said he wished he had the time and the members the patience to take the paper and separate from the chaff the good grains of wheat in it, and get the flour. He said that there are a few very valuable things and some historic statements which are reliable. There is a deal of fancy and much good, but more of rich classification. He objects to some of the terms used, saying he had yet to learn of any acid called "saccharic" acid, and that there is no such thing as "diseased nutrition."

Dr. Winder.—I must say I feel very thankful that this paper has been presented. It covers the modes and functions of animal life, and is one of the most important subjects that can be brought to our attention. There are some positions taken by Dr. Cutter in this paper which I consider very questionable. But I am not disposed to cavil on these points. I am disposed to take the paper as a whole, as one of deep research, study and reasoning, and permanent value. The most important bearing which this paper has upon our profession is the question of food stuffs as therapeutic agents, a subject fruitful of suggestion.

Dr. Walker, of New Orleans, begs me to recommend to this Association the use of lime water for teeth of poor structure, but I do not believe that it has any effect whatever. In the first place I do not find that the osseous system in lime-water districts is superior in any respect to the osseous development in districts not thus characterised. If I had any evidence that teeth were better in lime-water districts I would accept Dr. Walker's statement as true. The secretions of the mouth also have much to do with this question of dental caries. There is trouble everywhere. What is salivary calculus but these very earthy salts? I ask myself the question, would nature so lavishly throw away the substances which she so much needed? Do we not see mouths in which are teeth of poor structure full of calculus? As a matter of statistical evidence the people of Louisianna have the best teeth in the United States, and those in Massachusetts the worst, and the latter have abundance of lime-water, while there is none in Louisiana. I hold that you must have a power behind these salts to assimilate them.

Dr. Atkinson.—It is better for us to know a little and know it well than try to grasp a great many fanciful notions. What is food? We are told it is that which supports the organism. If it acts as food you must necessarily be healthy. When you become unhealthy, what is it? You have been poisoned. If you will take any substance that will correct and regulate, it becomes medicine, and medicine itself must be food. The subject was passed.

The president appointed as the committee to prepare communications for the papers of Washington and Baltimore, Drs. T. S. Waters, E. P. Keech and S. H. Williams, of Baltimore, and J. C. Smithe, R. B. Donaldson and J. B. Ten Eyck, of Washington.

Dr. W. H. Atkinson read the following paper on Histology and Microscopy:

The early records of histological knowledge originated from crude observations, made by the unaided use of the natural visual powers. From these arose the forms of physiological pronouncements in pathological formulæ known as Solidal, Humoral and Neural pathologies.

First. Solidal Pathology; or that based upon the supposition that function was the act of the solids of the animal body.

Second. Humoral Pathology; or that arising from the conception of the fluids of the body being the seat of functional activity.

Third. Neural Pathology; or that resulting upon a discovery of the nervous system as the channel through which function was induced. These three, so-called systems of pathology are all true in a sense as aspects of systemic or bodily performance of function.

Then, as now, men were apt to consider that which they perceived at the time to be all there was to be known. Hence the bitter contentions and controversies which mark the growth of Physiology in every age previous to the discovery, manufacture and use of the *Microscope*. So soon as this occurred a new field was opened up for better observations and closer discriminations than had been possible theretofore, (aside from inspirational methods of intensified minds.)

In seeking for the factors of function, generalizations and specializations become necessary to a classification adapted to the facts presented in the bodies under examination. And the bodies themselves must be subjected to methods of determining likeness and difference, so as to classify them in serial order to enable us to recognize shape, size, density, position, etc., etc., all of which conspire in fitting the parts to become the whole of the tissue, organ or body upon which we are making our study.

A wise use of the microscope is able to reveal the tissues, but not the elements of tissues. Ratiocinative methods only are adequate to the task of dealing with Mass and Energy, the confluence of which conspire to produce an unseen germinal outlay of tissual, organic, and systemic conformation, so beautifully revealed to the natural and microscopic vision in the study of Embryology.

Until 1872 *Protoplasm* was reckoned as an amorphous mass of indifferenced possibilities out of which tissues took their rise in some occult manner. After that date, no one conversant with the investigations of Carl Heitzman, now of 37 West 45th St., New York City, can longer exclude protoplasm from the category of formed and forming tissue; for he has proved its reticular (saccular) structure which every competent microscopist can verify for himself.

*Histology* is properly the *science* of weaving, or web-making, if we confine ourselves strictly to the legitimacy of the term. Therefore there must be the warp and the woof of which to weave the web of tissue, which is the material out of which webs and organs are constructed. This material is known as protoplasm, and is the resultant of complete digestion and admixture of food into an homogeneous—heterogeneity of substance. Just at what point of the digestory process we may be entitled to properly call it protoplasm requires very close discrimination to determine. In a general way the juices of the flesh have been called "pabulum," and "protenaceous compound," which is the prepared mass—substance from which the various tissues are derived, and by appropriations of portions of which they are nourished and kept in working order. So occult is this process of untried activity, that any tissue being debilitated or in need of nourishment (and in reach of protoplasm) will appropriate this same mass-substance without any loss by metamorphosis of the molecular mass; each element finding its special affinity awakened and engaged to build or nourish the weedy territory, without deficiency or excess of mass or energy, so that no debris whatever is present at the point of metamorphic exchange of the molecular mass as a whole or as a part of the bodies of which the corpuscles which go through the marvelous transformations into embryonic corpuscles—

protovertebræ—nerve-plates, muscle-plates and bone plates, from which are constructed the Nervous, Muscular and Osseous Systems. In fact all the other tissues of the whole body are but ripening and ripened careers of this one tissue protoplasm, without which we can have none of its derivatives.

*Epithelium* is nothing but slightly metamorphosed protoplasmic bodies, now called embryonal corpuscles. The three embryonal sheets known by the names Epi-blast, Hypo-blast and Meso-blast, are nothing else than continuous layers of these embryonal corpuscles folded in upon themselves so as to present us with clefts which are the beginnings of the Alimentary, Respiratory, Circulatory and Genito-Urinary tracts. The head fold, the tail-fold and yolk-fold present us with the body-walls and visceral-walls, the head-fold becoming the mouth, the tail-fold the anus, and the yolk-fold the umbilicus. All, as before said, without debris occurring anywhere until it is produced by the perfection of the digestory tract.

Dr. Winder.—Mr. President, the gentleman has left no room for criticism of his paper. He is too orthodox to leave himself open for criticism. For myself I should not like to attempt to criticise a paper by Dr. Atkinson on this subject. If there are errors I am not well enough informed on the subject to attack and expose them. I endorse it fully. There is in it an infinite amount of matter condensed in a very small space. I do know that Dr. Atkinson has been a close student on this subject for a great many years. The subject was then passed.

The sections then elected their chairmen with the following results:

*Sec.* 1. Surgical and Operative Dentistry:

Dr. J. B. Ten Eyck.

*Sec.* 2. Metallurgy and Chemistry of Metals:

Dr. R. F. Hunt.

*Sec.* 3. Artificial Dentistry: Dr. T. S. Waters.

*Sec.* 4. Dental Education and Literature: Dr. J. B. Hodgkin.

*Sec.* 5. Anatomy and Physiology: R. B. Winder.

*Sec.* 6. Histology and Microscopy: Dr. W. H. Atkinson.

*Sec.* 7. Pathology and Etiology: Dr. E. P. Keech.

*Sec.* 8. Diagnosis and Therapeutics: Dr. H. C. Thompson.

*Sec.* 9. Chemistry and Materia Medica: Dr. W. H. Atkinson.

*Sec.* 10. Not having reported, no chairman was elected.

The President announced clinics for the following morning and the meeting adjourned.

[TO BE CONTINUED.]

